# Development and Characterization of the Edible Packaging Films Incorporated with Blueberry Pomace

**DOI:** 10.3390/foods9111599

**Published:** 2020-11-03

**Authors:** Anika Singh, Yixin Gu, Simone D. Castellarin, David D. Kitts, Anubhav Pratap-Singh

**Affiliations:** 1 Food, Nutrition, and Health, Faculty of Land & Food Systems, 2205 East Mall, University of British Columbia, Vancouver, BC V6T 1Z4, Canada; anika.singh@ubc.ca (A.S.); yixingucara@gmail.com (Y.G.); david.kitts@ubc.ca (D.D.K.); 2 Wine Research Centre, Faculty of Land and Food Systems, 2205 East Mall, University of British Columbia, Vancouver, BC V6T 1Z4, Canada; simone.castellarin@ubc.ca

**Keywords:** blueberry pomace, active packaging, starch films, migration, sustainability

## Abstract

This work focused on the development of starch-based (potato, corn, sweet potato, green bean and tapioca) edible packaging film incorporated with blueberry pomace powder (BPP). The optical, mechanical, thermal, and physicochemical properties were subsequently tested. The film color was not affected by the addition of BPP. BPP incorporated into corn and green bean starch films showed increased light barrier properties, indicating a beneficial effect to prevent UV radiation-induced food deterioration. Film thickness and transparency were not primarily affected by changing the starch type or the BPP concentration, although the corn starch films were the most transparent. Furthermore, all films maintained structural integrity and had a high tensile strength. The water vapor transmission rate of all the films was found to be greater than conventional polyethylene films. The average solubility of all the films made from different starch types was between 24 and 37%, which indicates the usability of these films for packaging, specifically for low to intermediate moisture foods. There were no statistical differences in Differential Scanning Calorimetry parameters with changes in the starch type and pomace levels. Migration assays showed a greater release of the active compounds from BPP into acetic acid medium (aqueous food simulant) than ethanol medium (fatty food simulant). The incorporation of BPP into starch-chitosan films resulted in the improvement of film performance, thereby suggesting the potential for applying BPP into starch-based films for active packaging.

## 1. Introduction

At present, plastics are the most commonly used food packaging materials globally [1]. These materials are mostly non-reusable, non-biodegradable, and can, under specific conditions, lead to toxic outcomes and severe environmental consequences [2,3]. Recently, considerable research activity has been directed to finding alternatives to plastic packaging materials, mainly due to increasing consumer awareness toward individual health, food and nutritional quality, food safety and environmental sustainability [4]. As such, innovations in developing edible packaging films have resulted in environmentally friendly, non-toxic and biodegradable products [5]. These films are typically made from plant-based components, such as polysaccharides, proteins and lipids, or a combination of all [6]. Edible films and coatings are similar in composition, but coatings are applied on food surfaces by dipping, spraying, or brushing, while films are often thicker and free-standing [6].

Advances in designing edible packaging films have mostly used starch as a promising plant base material, with the capacity to carry antioxidant compounds. Examples include blueberry pomace powder (BPP), cocoa nib, tea leaf extract and essential oils [1,6]. Efforts have been made to include bioactive components in these films when enabling migration into food or the surrounding environment in the package (e.g., headspace) for extended shelf-life and improved food quality and safety [7]. The incorporation of antioxidants can provide food protection against oxidation, microorganism growth, enzymatic browning, and vitamin losses [8]. Furthermore, starch is low in cost and available from many plant sources, with proven excellent film-forming properties [9,10,11].

The industrial food manufacturing/processing industry is contributing to the increased agro-industrial waste that will jeopardize the environment. In particular, blueberry juice processing wastes, including skins and seeds, are typically discarded despite the fact that they represent a rich source in vitamins, minerals, fibers, and phenolic compounds, such as anthocyanins with antioxidant capacity and potential carry-over health-promoting effects [12,13]. Moreover, anthocyanins also change color when subjected to different pH conditions, a feature that could be useful when there are chemical–microbiological-induced pH changes occurring in food products with extended storage [14]. The incorporation of BPP into primary packaging formulations is a potentially viable alternative to produce a biodegradable, active and intelligent food packaging material, while also producing environmentally friendly products that contribute to reduced waste generation. The addition of BPP into edible packaging films shall provide an alternate source for valorization of the blueberry industry waste stream, as well as the added benefits of migration of antioxidant compounds into the food products that shall increase their nutritional and health benefits.

The objective of this study was to develop and characterize different starch (corn, potato, green bean, sweet potato, and tapioca) sources for developing edible films with BPP that could be used in food packaging. For this purpose, we tested different starch sources with varying BPP concentrations to evaluate the effect on film performance, in particular, thermal and optical properties, as well as physicochemical behavior. The release of phenolic compounds into simulated food matrices was subsequently tested using migration assays.

## 2. Materials and Methods

### 2.1. Materials

A freeze-dried blueberry pomace powder (BPP) was obtained from Pacific Coast Fruit Products Ltd. (Vancouver, BC, Canada). Corn starch was purchased from ACH Food Companies, Inc. (Memphis, TN, USA). Potato and Tapioca starch was supplied by Yi-Feng Food Co., Ltd. (New Taipei, China). Green bean starch was acquired from Paojung enterprise Ltd. (Richmond, VA, Canada). Sweet potato starch was obtained from Sichuan Youjia Foodstuffs Co., Ltd. (Chengdu, China). Sorbitol was purchased from VWR International Co. (Mississauga, ON, Canada) to be used as a plasticizer. Chitosan (85% deacetylated) was purchased from VWR International Co. (Mississauga, ON, Canada).

### 2.2. Film Preparation

The film preparation was performed according to the method published by Bourtoom and Chinnan [15], with minor modifications. In short, a film-forming solution was prepared by dissolving 4g of respective starch (corn, potato, tapioca, green bean and sweet potato) in 100 mL distilled water and heating it at 90 °C for 30 min with constant stirring. The mixture was then cooled to 27 °C. Another solution of chitosan (2 g/100 mL) was prepared in acetic acid (1% *v/v*). The dispersed chitosan was stirred for 30 min, or until all the chitosan was dissolved. In the next step, starch–chitosan blend films were prepared by mixing 100 mL of the starch and chitosan solution into each other, along with three levels of blueberry pomace (0-control, 0.4 and 0.8 vol%) with heating at 90 °C and constant stirring for 30 min. Sorbitol was used as the plasticizer at a concentration of 30% [15]. To make the film, the respective film-forming solution was poured into a petri dish and allowed to dry at 50 °C for 8 h. The films were removed and conditioned at 64.4% RH using a saturated solution of NaNO_2_ at 25 °C for at least 48 h in a desiccator. Each experiment was performed in triplicates. Film thickness was measured to the nearest 0.01 mm with a hand-held micrometer. The values obtained for each sample at ten different locations were averaged.

### 2.3. Color and Texture Analysis 

Film color was measured using Hunterlab LabScan XE (Hunter Associates Laboratory, Inc., Reston, VA, USA). A white standard plate (Standard No. LX17505) (calibration plate values: X = 80.09, Y = 84.98, and Z = 89.67) was used as a background for film color measurement. The color parameters ware measured using the CIELAB color scale, and total color change was calculated with the following formula, where *L* = 0 (black) to *L* = 100 (white), −*a* (greenness) to +*a* (redness), and −*b* (blueness) to +*b* (yellowness):

(1) $$\Delta E=\sqrt{{\left(L_{\mathrm{control}}-L_{\mathrm{sample}}\right)}^{2}+{\left(a_{\mathrm{control}}-a_{\mathrm{sample}}\right)}^{2}+{\left(b_{\mathrm{control}}-b_{\mathrm{sample}}\right)}^{2}}$$

where *L*_control_, *a*_control_ and *b*_control_ refer to the *L*, *a* and *b* values of respective starch films without the addition of blueberry pomace.

The tensile strength (hardness) of the films was obtained using TA XT plus texture analyzer (Texture Technologies Crop, New York, NY, USA) equipped with a tensile grip (TA 96B) attachment [16]. Films were cut into rectangular strips (5 mm width × 50 mm length) and loaded with an initial grip separation of 20 mm, and a pre-test and test speed of 1 mm/s. All the films were conditioned, as described in Section 2.2, before testing.

### 2.4. Ultraviolet-Visible (UV-Vis) Spectroscopy

The transparency value of the developed film was assessed using absorbance. A TECAN Infinite ^®^ M200 pro-UV-vis spectrometer (Tecan Systems Inc, San Jose, CA, USA) was used for obtaining UV-vis spectroscopy values in the range of 230–800 nm. Films were cut into small sizes and were placed in a microplate with no air gap between the film and the plate. Three samples were measured from each type of film. The empty walls were also measured to be used as the blank. The film’s transparency value was then calculated using Equation (2), where *A*_600_ is the absorption at 600 nm, and ‘*x*’ is the film thickness.

(2) $$\mathrm{Transparency}\;\mathrm{value}\;=\frac{A_{600}}{x}$$

### 2.5. Gloss Measurements

The relative gloss of each film was measured at 60° incidence angle three times using a Gloss meter (ETB-0686 Glossmeter, 60°) purchased from M & I Instruments Inc. (Mississauga, ON, Canada). This angle was determined to be optimal [8]. The gloss values were measured on both the support side (surface in contact with the non-stick tray during film drying) and the air side (surface exposed to air during drying) of the developed films. All results were expressed as gloss units, relative to a highly polished surface of black glass standard, with a value near to 110. A total of six measurements were taken on each sample.

### 2.6. Differential Scanning Calorimetry (DSC)

DSC measurements were performed using a PerkinElmer DSC 4000 (PerkinElmer, Akron, OH, USA). One milligram of the sample was hermetically encapsulated in aluminum capsules and was placed in the sampling unit. The sample was heated from −30 °C to 270 °C at a rate of 10 °C/min under the nitrogen atmosphere.

### 2.7. Water Solubility

Films were cut into 2 × 2 cm^2^ pieces, dried at 105 °C for 24 h, and weighed. Each piece was placed in a 50 mL beaker containing 20 mL of distilled water, and the vessel was capped and stored at 25 ± 1 °C for 24 h. Film samples were removed and dried at 105 °C for 24 h and weighed to determine the final dry matter. The percent solubility of the film was calculated and expressed as a percentage of the initial dry matter content in Equation (3) [9].

(3) $$\%\mathrm{Solubility}\;\left(\mathrm{DB}\right)=\;\frac{\left(\mathrm{Film}\;\mathrm{dry}\;\mathrm{mass}\;\mathrm{before}\;\mathrm{test}-\mathrm{Film}\;\mathrm{dry}\;\mathrm{mass}\;\mathrm{after}\;\mathrm{test}\right)}{\mathrm{Film}\;\mathrm{dry}\;\mathrm{mass}\;\mathrm{before}\;\mathrm{the}\;\mathrm{test}}\;\times \;100\%$$

### 2.8. Water Vapour Transmission Rate (WVTR)

**Figure 1:**
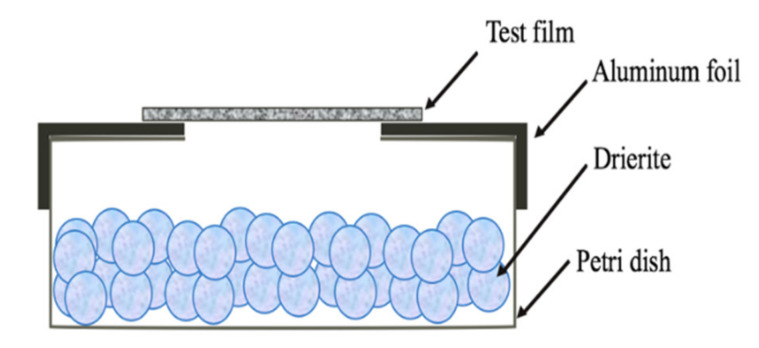
Set-up to study the water vapor transmission rate from developed flexible packaging films.

The film WVTR was determined gravimetrically, according to the method described by Hu et al. [17] with some modifications. First, a 2 cm × 2 cm opening was cut on a piece of aluminum foil. The foil was then used to cover the opening of a petri dish, and the edges were sealed by parafilm. This left a 2 cm^2^ opening on the apparatus. Then, 7.50 g ± 0.05 g drierite was weighed into the aluminum foil-covered petri dish. Next, the films were cut into 3 cm × 3 cm squares, and the edges were glued on the aluminum foil to create 0% RH inside the petri dish. The assembled apparatus was placed in a desiccator for 24 h to let the glue dry. A diagram of the finished apparatus is shown in Figure 1. The apparatus was always checked for proper seal before use.

When the apparatus was ready to be used, it was weighed and immediately put into a 64.4% RH environment created by another desiccator with a saturated solution of NaNO_2_ at 25 °C. The weight of the whole apparatus was recorded initially every one hour, six hours, and also at least one time between seven and twelve hours. Then, the slope of the weight gain as a function of time normalized to the testing area was taken as the WVTR (Equation (4)).

(4) $$\mathrm{WVTR}\;=\;\frac{\mathrm{Mass}\;\mathrm{of}\;\mathrm{water}\;\mathrm{gain}}{\mathrm{Time}\;\times \;\mathrm{Area}}$$

### 2.9. Migration Analysis

EU 2016/1416 protocols were used to perform migration analysis using a total immersion migration test, wherein 96% ethanol and 3% acetic acid were chosen as food simulants for fatty and aqeous foods, respectively. A 6 cm^2^ piece of each film sample was immersed in 10 mL of the respective ethanol and acetic acid food simulants, which were then placed in an oven at 20 °C for 10 days. UV-vis measurements were performed on the simulant solutions at the end of the study using a TECAN Infinite ^®^ M200 pro-UV-vis spectrometer (Tecan Systems Inc, San Jose, CA, USA).

### 2.10. Statistical Analysis

Analysis of variance (ANOVA) was used to determine the significance of differences among the samples. Differences were statistically significant at the *p* < 0.05 level. Fisher’s LSD (least significant difference) test was used to determine significant differences among treatment means. Some parameters were also subjected to the Pearson correlation coefficient procedure. Data were analyzed using Microsoft Excel (2010).

## 3. Results

### 3.1. Optical and Physical Properties 

#### 3.1.1. Color 

**Figure 2:**
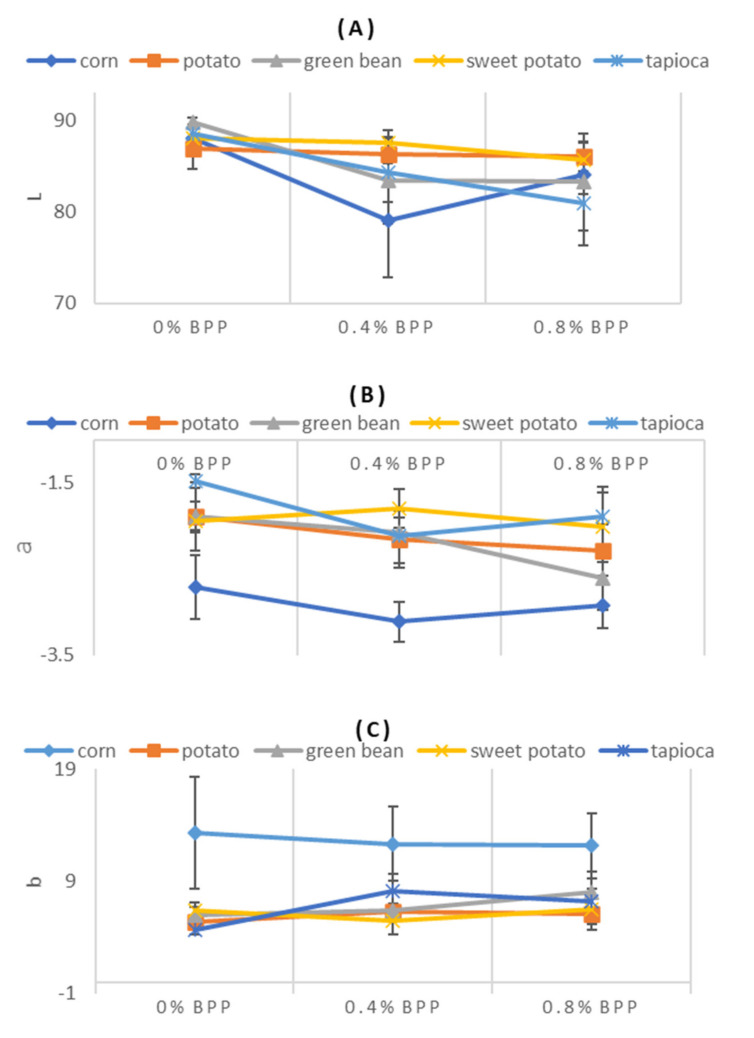

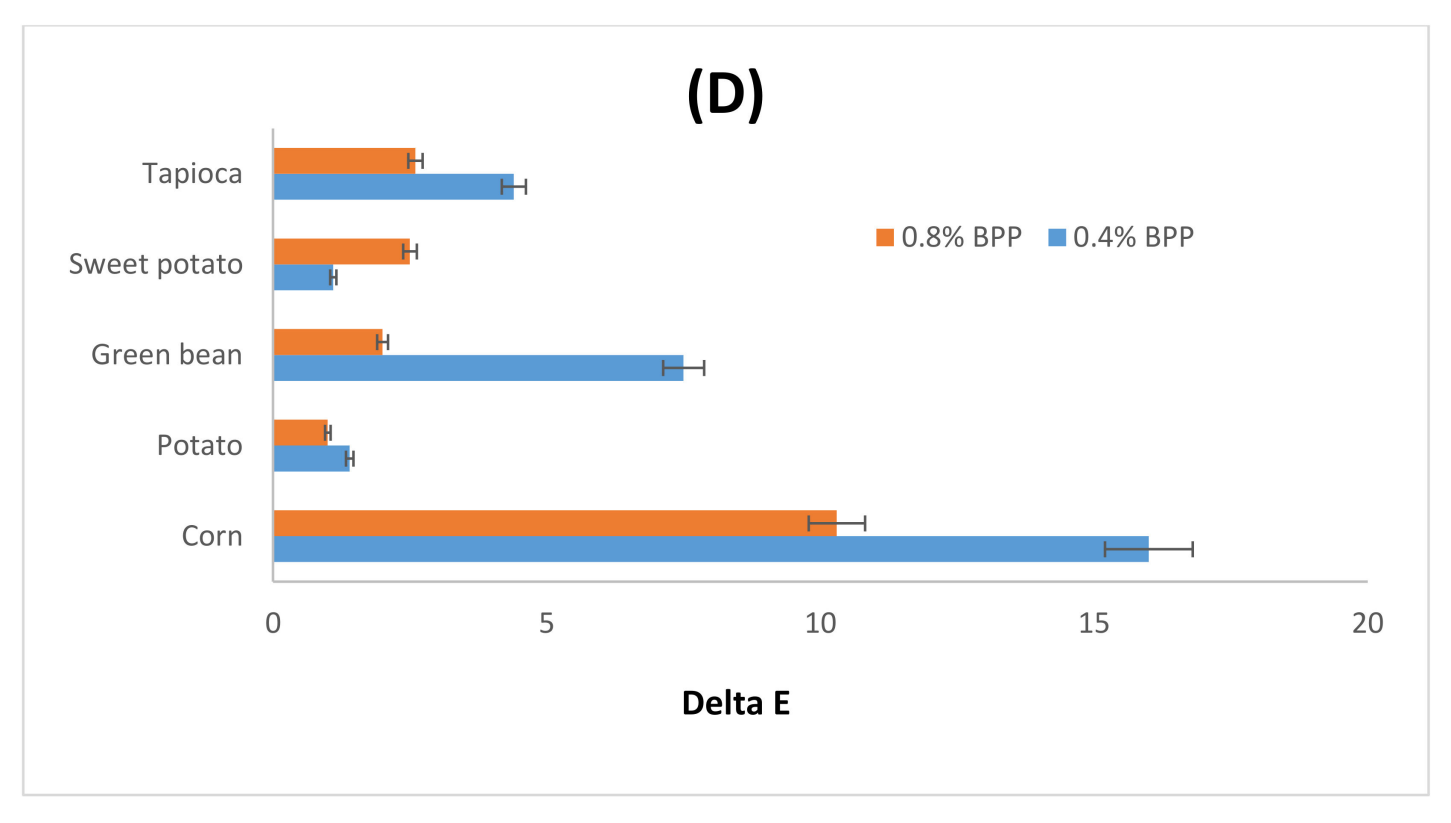
The *L* (**A**), *a* (**B**), *b* (**C**) and total color difference (**D**) values of the developed films made with 0%, 0.4% and 0.8% BPP with each starch type. Data points represent the mean of ten measurement and error bars denote standard deviations.

The average of *L*, *a*, and *b* values of all the films are shown in Figure 2. The *L* values (Figure 2A) of the films were not significantly affected by the use of different starch sources, while values *a* and *b* were significantly different (*p* < 0.05) for the corn starch derived films. The *a* value of corn starch film was lower than the other four starch films. (Figure 2B). Similarly, the *b* value of corn starch film was higher than in other films (*p* < 0.05) (Figure 2C).

The *L* values, in general, showed a decreasing trend with increasing BPP, including corn starch film, which showed a substantial and significant reduction in the *L* value when BPP was changed from 0% to 0.4%. At the 0.4% BPP level, the corn starch films showed the lowest *L* value (*L* = 79.08). This trend was, however, reversed (only with corn starch films) when BPP was further increased to 0.8%. All the films had a negative *a* value, which represents slight greenness in the developed films. It should be noted that the *a* value of corn starch films was significantly lower than the rest at 0% and 0.4% BPP level (*P* < 0.05). This trend was continued when the BPP level was changed to 0.8%, although the difference was not significant. There was no significant change in *a* value when the BPP concentration in the film varied. The maximum *b* value was 13.31 for corn starch films, while the minimum was observed for tapioca (*b* value = 4.69) at 0% BPP. The positive *b* values indicated a slight yellowness in all developed films. The BPP level contributing to a slight increase in the *b* value was noted at higher BPP levels only. 

The assessment of the overall change in the color of developed films (Δ*E*) is presented in Figure 2D. The Δ*E* value is a useful way to describe the degree of difference in color change that is visible with the human eye. For example, a Δ*E* higher than 3 represents a color difference perceivable by the human eye [18]. The maximum value of Δ*E* for corn starch films was obtained with 0.4% BPP. The Δ*E* value in the rest of the films varied between 1.1 and 7.5 (Figure 2D).

#### 3.1.2. Ultraviolet–Visible (UV-vis) Spectroscopy

The total UV absorbance of samples was calculated as the area under the net absorbance curve from wavelength 230 to 400 nm (Table 1). From this, the maximum total absorbance (at 0.8%BPP) for corn films (164.1 ± 1.7) was not different from green bean (150.9 ± 1.6). Similarly, the three starch films, sweet potato, potato and tapioca, were also not different from each other. 

For all films developed in our study, the peak UV absorption was recorded between 287 nm and 297 nm. The total UV absorption of corn starch films was significantly (*p* < 0.05) affected by the level of BPP. For example, films containing 0.8% BPP had significantly (*p* < 0.05) higher UV absorption than those containing 0.4% and 0% BPP films, respectively, with no substantial difference found between the latter two. Peak UV absorbance for potato starch films was obtained at 289 nm, and the results showed that potato starch films with added BPP had a significantly higher total UV absorbance than those without BPP (*p* < 0.05). Green bean starch films displayed a trend of increasing total absorbance with the addition of BPP. The lowest total absorbance recorded at 0% BPP level was equivalent to a 33.3% reduction in absorbance. 

The maximum absorbance (72.7 ± 3.8) for sweet potato films was obtained at the 0.8% BPP level, in contrast to minimum absorbance (48.3 ± 3.8) when 0.4% BPP was added. Although a slight decrease in total absorbance was recorded when the BPP level was changed from 0% to 0.4%, the effect was not statistically significant. Increasing the BPP in tapioca starch films produced no change in total absorbance. In general, potato starch films were influenced the least by the added BPP levels.

#### 3.1.3. Film Thickness and Transparency

The thickness of the developed films varied between 0.09 mm (green bean) and 0.13 mm (Tapioca) (Table 2). In the absence of BPP, the difference between various starch-based films was not significant; however, tapioca films were, in general, notably thicker than all. The average transparency values of films made from each starch type are given in Table 2. The highest transparency was observed for corn starch films with no BPP added (1.02 ± 0.05). Transparency values are inversely related to the transparency of the film [1]. The presence of BPP in the film did not influence the transparency of all the films, with one exception being the corn starch films, where the transparency value was significantly reduced (*p* < 0.05) by 26–30% when BPP was added. 

#### 3.1.4. Film Gloss

The impact of starch type on film gloss on the support surface was significant (*p* < 0.05) (Table 3). When BPP was not added, tapioca (74.1 ± 2.8) films had the highest gloss values, whereas others were not significantly different from each other. On the air side (e.g., the surface exposed to air during drying), the starch type had a significant effect on the gloss of films. Green bean (66.6 ± 3.7) and potato (54.0 ± 1.8) starch films had the highest gloss values, followed by tapioca (38.5 ± 1.4), sweet potato (34.3 ± 9.1) and green bean (18.6 ± 1.6) starch films.

The gloss observed on the air side of starch produced films, such as sweet potato and tapioca, were not significantly different from each other. The corn starch film had the lowest gloss among all films. Except for sweet potato starch films, significant differences existed between both the support and air side, respectively, for film types when no BPP was added. The addition of BPP resulted in no differences between either the support side or the air side, respectively, in green bean, sweet potato and tapioca films. Moreover, in general, the air side of all the films had greater gloss than the support side.

Only the support side of corn and tapioca starch films and the air side of potato and tapioca films were significantly affected by the addition of BPP. Similarly, the air side of corn starch films showed no difference in gloss when BPP was added. The addition of BPP to potato starch films had no effect on the gloss related to the support side of these films, while the same significantly helped increase gloss on the air side of the film.

#### 3.1.5. Tensile Strength (TS)

**Figure 3:**
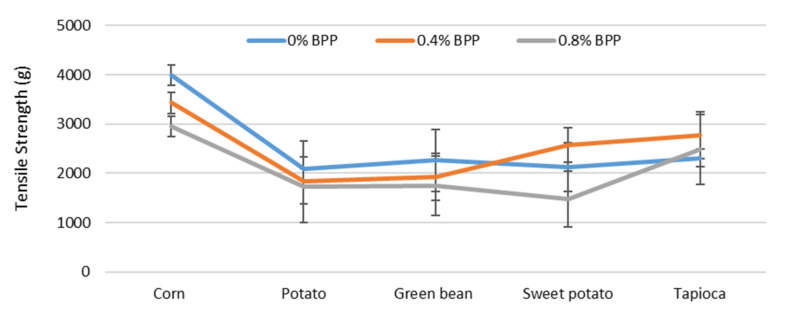
The tensile strength of films made from different types of starch and BPP levels. Data points represent the means ± standard deviations of six replicates (unit: g).

The average TS for films produced from each type of starch is shown in Figure 3. In the absence of BPP, the TS values of all the films, with the exception to corn starch, were not significantly (*p* < 0.05) different. Corn starch films, which had the highest relative TS of all starches produced, were not compromised when BPP was present. Increasing BPP content in the corn starch film lowered TS. Similar trends were observed for sweet potato and green bean starch films; however, the reduction in TS value was not related to the BPP level. For tapioca starch films, no significant change was noted with BPP.

### 3.2. Physiochemical Properties (WVTR and Solubility)

When no BPP was added, the potato starch films produced the lowest (*p* < 0.05) WVTR, while the maximum WVTR was recorded with tapioca starch films (*p* < 0.05). (Table 4). Increasing the BPP level had no significant effect on WVTR in different films, with the exception of the tapioca film, which showed increased WVTR at 0.8% BPP. 

The solubilities of films made from different starch sources were not different. Furthermore, adding the BPP level had no effect on film solubility, with the exception of a slight change in green bean starch films. Green bean starch films containing 0.4% BPP were significantly more soluble than when BPP was present at 0.8%. 

### 3.3. Thermal Properties

**Figure 4:**
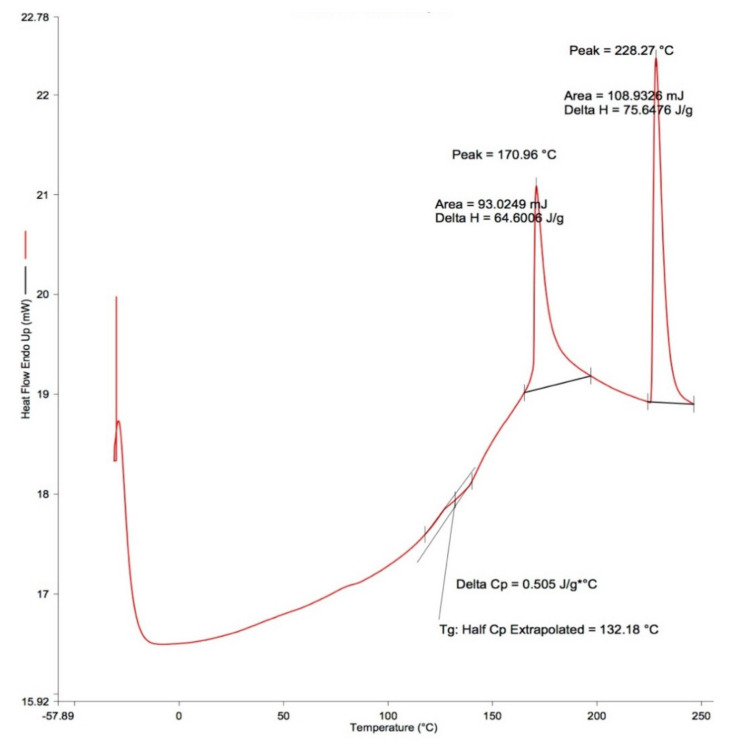
The differential scanning calorimetry (DSC) result of corn starch film without blueberry pomace (BPP) added.

DSC measurements were performed on all starch gels, and thermograms are displayed in Figure 4. The five DSC parameters reported herein on films were as follows: (a) glass transition temperatures (Tg); (b) enthalpy of the first (Δ*H-1*) and the second peak (Δ*H-2*); and c) the melting temperatures of the first (Tm-1) and second (Tm-2) peak (Table 5). All films displayed two distinct peaks on the DSC curves. The Tm-1 of all the films ranged between 168 °C and 180 °C, whereas the Tm-2 were between 197 °C and 243 °C. The starch type had no effect on DSC parameters.

The presence of BPP had no effect on Tg and Δ*H-1*, Tm-1 or Tm-2 for all films. The only exception was with corn and green bean starch films, where Δ*H-2* was significantly (*P* < 0.05) affected by BPP levels. These films with low BPP content (e.g., 0% and 0.4%) showed higher Δ*H-2* than their counterparts containing 0.8% BPP.

### 3.4. Migration Assays

**Figure 5:**
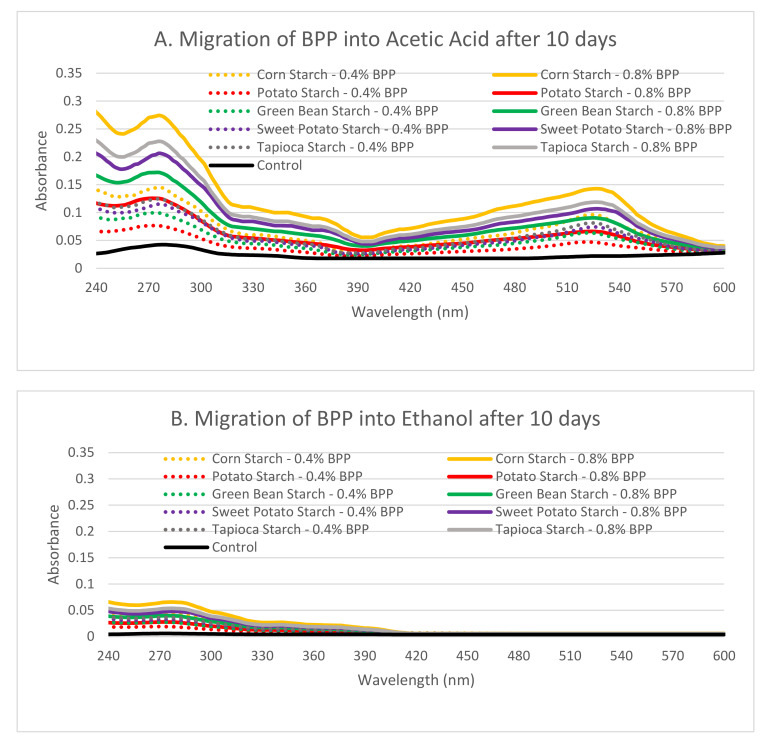
UV absorbance curves of the simulant solutions after extracting corn, potato, green bean, sweet potato and tapioca starch films with different contents of blueberry pomace (BP) in (**A**) acetic acid and (**B**) ethanol.

In vitro migration assays were performed to assess the migration of BPP compounds into food-simulating solutions (fatty food—ethanol; aqueous food—acetic acid). No discolorations were observed, and the films remained intact over the 10 day migration assay. The color of the simulating solutions changed progressively over the 10 day period, with films dipped in aqueous simulant (acetic acid) showing a more pronounced red color than films immersed in fatty food simulants (ethanol). The UV-vis absorption spectra of the respective simulants after immersion in corn, potato, green bean, sweet potato and tapioca starch films are shown in Figure 5, wherein it was observed that the corn starch film showed the highest peaks at 280 nm and 530 nm at increasing concentrations of BPP, followed by tapioca, sweet potato, green bean and potato starch films, respectively.

## 4. Discussion

### 4.1. Color

Former studies have shown that starch characteristics, such as the amylose/amylopectin ratio, granule size and granule shape of different starch sources, can influence color [9]. In the present study, we report that the different starch sources used to generate films were not a factor for the color of the packaging materials. Specifically, the *L* values of different films made from different starch sources indicated no difference in lightness. The corn starch films exhibited higher *b*-values or a greater yellow tone compared to others. We attribute this observation to the possible formation of early Maillard reaction products that would have formed due to the drying temperature used in this study [19]. This may have a negative effect on consumer acceptance; hence drying temperatures should be optimized to prevent color development on corn starch film surfaces. Adding BPP to the starch sources generally decreased lightness, a finding that was reported when apple pomace and green tea extract were added to the starch-based packaging films [20]. The greenness (*a* value) of films with BPP was slightly higher than those without BPP, which is inconsistent with previous findings [1]. Luchese et al. (2018) showed that adding BPP to starch films promoted the formation of radish color [1], which we did not observe in our study. One explanation could be that they used higher concentrations of BPP, and the BPP may not have been homogenized, thus contributing to inconsistent color measurement. Overall, all the starched-based films (except corn starch) were affected the least by varying BPP levels, which indicates that this particular starch source was a superior choice to make films when color is an essential parameter of the film. Finally, corn, green bean and tapioca starch films were found to have Δ*E* > 3, at varying BPP levels, thus indicating that the incorporation of a small amount of BPP could also result in color differences.

### 4.2. UV-Vis

An effective packaging film must protect against UV deterioration; it is required to have high UV absorbance. Our results showed that the absorbance of corn and green bean films added with BPP was significantly higher than other starch sources tested, which indicates a potential for better UV protection. We only used 4% starch in our study, even though others have achieved better light barrier properties using a higher starch concentration [21]. The UV maximum absorption peaks for all films were between 250 nm and 300 nm, which implies that the films could provide an extended shelf life of products that are susceptible to photo-oxidation [19]. The general trend of increasing UV absorbance with the presence of BPP in films can be attributed in part to the anthocyanin mixture. Potato and tapioca films did not follow the same trend, probably because of the non-uniform thickness of the film, and samples might be taken from thicker areas, which made them a better barrier to light [21]. Luchese et al. (2018) observed a similar finding that was identified at a longer wavelength than the shoulder structures formed at around 280 nm, due to the presence of aromatic compounds in cassava starch films [1]. The absence of the shoulder structure might be due to the BPP levels used herein, as compared to those employed by Luchese et al. (2018) [1]. The potato, sweet potato and tapioca films showed no significant change in absorbance at high BPP concentrations. Future research should explore this more by using a higher BPP level added to these starch films than what was employed herein. One exception was with the tapioca starch films, where different BPP concentrations gave similar absorbance curves, thus indicating that tapioca starch films without BPP were equally efficient in protecting against UV deterioration compared to 0.4% and 0.8% BPP, respectively. 

### 4.3. Thickness and Transparency

It was expected that using starch with a higher amylose content would result in a thicker film [9]. However, in our study, no significant differences were found between the thicknesses of films made from different types of starch. We attribute this to the fact that only small differences existed between the different starch sources used in our study for amylose content. Further, the incorporation of BPP at concentrations used herein did not significantly change the thickness of starch films. This result contrasts other findings in which the addition of antioxidants in films from higher initial starch concentrations produced a thicker film, despite the constant amount of solid per surface unit [8]. 

The transparency value corresponds to a higher visible light absorbance, which indicates the lower transparency of the film [22]. Our findings with corn starch agree with previous studies [9] where films derived from corn starch were opalescent, compared to transparent potato starch films. Generally, a higher transparent film is preferred by consumers; however, low transparency does not always indicate the inferior quality and may not protect the food as well from photo-oxidation-induced lipid oxidation [22]. Therefore, corn starch films were considered less desirable when high transparency is needed, whereas on the other hand, it may also have an advantage in preserving food prone to light deterioration. 

Research has shown that starch granules that are mainly composed of semi-crystalline amylose can scatter a portion of the light, which results in lowering the transparency [21]. Hence starch with low amylose content is expected to be more transparent. In edible starch films, the amylose/amylopectin ratio is proven to be a factor significantly affecting the physical and chemical properties. This ratio defines the microstructure and viscosity of the film-forming suspensions. The film thickness and hence the transparency are strongly affected by this ratio films [9,23]. Indeed, potato starch films were transparent compared to corn starch films. Although the transparency of films in this study generally followed the order of amylose level, this was not always the case (green bean starch had the highest amylose content, yet maximum transparency). This exemption might be explained by the variation in the film formulations and amylose contents of different starch cultivars [24]. Only corn starch films’ transparency was significantly affected by the BPP level; generally, the higher the BPP level, the lower the transparency. This is consistent with earlier findings, where the presence of green beans lowered the transmittance and gave higher opacity values due to increased turbidity [25]. Gutiérrez and Alvarez (2018) also reported that thermoplastic starch films devoid of an antioxidant source had the most excellent transparency [26]. We suspect that the fact that the incorporation of BPP made the film matrix less compact also resulted in it being a more heterogeneous film, and capable of providing greater light dispersion [9].

Our findings also lead to our conclusion that the transparency of films was more dependent on the starch source and the BPP concentration than the film thickness, which agrees with Kampeerapappun et al. (2007) [27], but is different from other findings [9,28]. This disparity of observations may be due to the low concentration of chitosan used in our 4% starch films. Others have reported that chitosan can reduce the transparency of biopolymer films when added at a higher level of concentrations (e.g., 15) [29]. Therefore, the effect of chitosan might also represent a variable to consider when determining the influence of thickness and the correlation between transparency and thickness. 

### 4.4. Gloss

Except for potato and tapioca starch films, other films indicated a higher roughness (<70 GU). Film roughness is required to improve the adhesion of inks on the film surface for printing and to enable adhesion between surfaces [1,30]. Corn starch films had the lowest gloss on the air side among all films, making it easier to be used for printing and heat-sealing [1]. It is essential given that gloss value is also a function of the amount of chitosan added to the film, which in our study was kept constant for film making. This also enabled compatibility between the two polymers, as shown by the DSC results, because phase separation will lead to higher surface irregularities and roughness, which will lower gloss [8]. On the support side, only corn and tapioca starch films had gloss values that were affected by the addition of BPP. Generally, the lower the BPP content, the higher the gloss of the film, which agrees with Luchese et al. (2018), who reported that the incorporation of BPP decreased the gloss of cassava starch films [1]. In a similar study, Gaikwad et al. (2016) reported that the addition of apple pomace led to a rougher surface without cracks, and the roughness increased as the amount of apple pomace increased [20]. Taken together, these findings support our results that the incorporation of BPP would also improve the film performance in printing and heat-sealing on the support side. However, the air sides of potato and tapioca starch films with 0.8% BPP had shown to have the highest gloss. This did not agree with the scanning electron microscopy results of polyvinyl alcohol films, in which the apple pomace showed a protruded film structure [20]. This protruded film structure was thought to increase the surface roughness and decrease the film gloss. Further investigations are needed to confirm this result or find possible explanations.

Generally, the smoother the surface of the film, the greater gloss it will show. Although the air side of the film was expected to have less gloss depending on the source of the starch material, when BPP was present, this was not the case for most of the films. Basiak et al. (2017) found that the corn and potato films were glossy on the support side and dull on the air side [9]. Our results agree with this for the corn starch films, but not with the potato starch films.

### 4.5. Tensile Strength (TS)

Ideally, the tensile strength of the films should withstand the normal stress encountered during the packaging, shipping and handling of the food in order to maintain integrity and barrier properties [8]. A significantly high TS associated with corn starch films in our study does not agree with other findings where the lower amylose contents of starch resulted in higher TS of films [9]. The importance of this variable is best seen with the different behaviors of starch films that are attributed to gelation, crystallinity and film-forming capacity. The difference in results can be explained by the different formulations used in this study, with the addition of chitosan and using sorbitol instead of glycerol as the plasticizer. Others found that wheat starch and chitosan were compatible polymers that form a homogeneous structure that increases the TS of films [8,15,31,32,33]. Sorbitol can also increase the TS of starch films more than glycerol, due to the higher number of hydroxyl groups attached, which will interact with the film matrix, thus limiting mobility and interactions with water [21]. BPP concentration did not significantly influence the TS of all films tested, which is in agreement with Gaikwad et al. (2016), who also reported that the TS of a polyvinyl alcohol film was not significantly altered with the incorporation of apple pomace at a concentration lower than 5% starch [20]. At those BPP levels, the relatively higher molecular weight of phenolic compounds, such as the procyanidins and anthocyanins present in the BPP, as well as hydrophilic properties of BPP, would contribute to the inter-chain interactions and similar TS of starch–chitosan films [34]. At higher concentrations, the presence of sugar in the BPP materials would also act as a plasticizer and contribute to a noticeable change in the mechanical properties of the film [35]. The insignificant correlation between TS and film thickness indicated that the influences of the starch level on TS were related more to the film materials than its thickness. Basiak et al., (2017), however, reported that the mechanical resistance of a film can be related to both thickness and water content [9]. 

### 4.6. Physiochemical (WVTR and Solubility)

A low WVTR in the starch film is desirable, as films are expected to limit the moisture transfer between the food in the package and the environment [11]. The average WVTR of all the films developed in our study was 100 times higher than pure low-density polyethylene (LDPE) film, which can be attributed to the fact that polyethylene films are highly hydrophobic and have relatively low permeability to water vapor [36]. However, compared to glycerol-plasticized cassava starch films with 1 wt. % chitosan coating, the WVTR of the starch–chitosan films made in our study was lower [31]. Our tapioca starch films had higher WVTR, which is contrary to previous findings that reported that starch with a lower amylose content will also have lower wettability and moisture absorption [9], which leads to a lower WVTR. Further, WVTR was also believed to be related to the ratio between crystalline and amorphous amylose and amylopectin components, and related polymeric chain mobility [37]. Therefore, the similarities in the WVTR of all films produced in this study indicated that the ratios between crystalline and amorphous zones and polymeric chain mobility were similar. There was no noticeable influence of BPP levels on the WVTR of films, which agrees with previous findings by Borah et al. [38]. However, they also reported that the decrease in WVTR caused by a denser matrix was counteracted by the larger pores formed, and the WVTR remained at similar levels. The lack of a correlation between solubility and thickness is evidence that the similarities between the WVTRs of different films were strongly related to the effects of starch and BPP levels. 

The low water solubility of the film is required to maintain the food integrity and resistance to water permeability in food packaging applications. However, for other applications such as food coating and encapsulation, water solubility is useful [9]. The average solubility of all the films made from different starch types was between 24 and 37%, which indicated that foods with low to intermediate moisture content are suitable for packaging with these films. The solubility of starch–chitosan films made herein was lower compared to glycerol-plasticized corn starch films (44.8%) [9]. The insignificant difference between the solubility of different starches indicated that there is little concern when choosing between the five types of starch used in our study [11]. 

Only green bean starch films had noticeably different solubility levels at different BPP levels, probably because the addition of BPP resulted in a packed film structure, which decreased its accessibility of water [38]. Overall, BPP did not primarily affect the solubility of the films either, probably because of the relatively low amounts of BPP incorporated. No significant correlation was found between solubility and thickness, so the difference in solubility of the film is more related to the properties of the film matrix than thickness. 

### 4.7. DSC Thermogram

We observed two peaks from the film DSC thermogram that indicated a multiphase system with intermixing of phases. The Tm of developed films without BPP varied between 80 °C and 100 °C, with a thermogram shift corresponding to the addition of BPP. The Tm of pure chitosan powder was 200 °C, and this compares with chitosan films made with an acetic acid solution that has a peak at 240 °C [39]. The first peak (between 168 and 180 °C) corresponded to the starch component and shifted towards the Tm of chitosan due to the intermixing of components. The second peak (between 197 °C and 243 °C) was attributed to chitosan. Other researchers have reported only one peak in starch–chitosan films [40] and starch–antioxidant films [1,41]. Within the experimental range used in our research, choosing any of the five starches did not affect the thermal properties of the film. However, others have discussed how the starch amylose/amylopectin ratio could affect the film Tg, because the Tg of amylose is higher than that of amylopectin [42]. This phenomenon may also explain the carbohydrate chain–chain interactions and induced-partial crystallinity by linear chains, as compared to branched chains [42]. 

The characteristic Tg of the starch film reflects its physical properties and stability to chemical and microbial activity. The starch level had no impact on Tg, and a single Tg indicates the good compatibility of the component polymers [22], indicating excellent compatibility between the different types of starch used herein and chitosan. Moreover, the Tg obtained also implied a similar flexibility character for all films. Food products kept at temperatures lower than Tg display greater stability over long storage periods than those stored at temperatures higher than the Tg [43]. All the films made in this study had a Tg higher than 120 °C, which indicates that the films developed in this study are relatively stable under usual food storage temperature conditions. However, package-on heat processing involving a higher temperature than the Tg are not as suitable for starch film-packaged foods. 

The enthalpy values (H) are an indicator of the loss of molecular order due to the disruption of hydrogen bonds. The enthalpy change (Δ*H*) was higher in our starch films than in cassava starch films [1]. The interaction and formation of strong physical bonding between the hydroxyl groups of starch and chitosan explains the higher Δ*H* [44], and is preferred. In specific cases, such as with sweet potato starch films, we observed an opposite trend in Δ*H*. Corn and green bean starch films with added BPP exhibited greater enthalpy than control film. Hydrogen bonds/other weak bonds stabilizing the network might be lower in those BPP added films. It is possible that the increased repulsion force between molecules resulted in the low enthalpy values used for breaking those bonds. Since the peak observed during the first heating cycle was highly influenced by the presence of water in the sample, we expect that different sources of the starch level will influence *ΔH^1^* if the humidity of the environment is not controlled. This could explain the differences observed between potato and sweet potato starch films, where different water contents in these starches exist [45].

Melting temperature (Tm) is an indicator of atomic bonding strength and water content [28,40]. The melting temperature of our films was higher than 200 °C. Increases in film Tm occur with higher starch concentration as the bonding strength between materials is also higher. A film with a high starch concentration would therefore have lower water contents due to the formation of a denser film matrix [28,40]. 

The level of BPP added to starch had no effect on the five thermal parameters of our films, with the exception of the corn starch films. The higher Δ*H-2* of these films indicated that the crystalline structures had greater thermal and chemical stability than amorphous structures. Moreover, the higher Δ*H-2* obtained for corn starch films with low BPP content indicated that adding BPP at concentrations up to 0.4% decreased film crystallinity.

### 4.8. Migration of Active Components of BPP Starch Films into Aqueous and Fatty Food Simulants

Based on in vitro analysis of the migration of the BPP incorporated starch films, it was observed that the simulating solutions started becoming reddish progressively over 10 days of storage. While a notable red color was observed for aqueous simulant acetic acid, fatty acid simulant ethanol showed only slight coloration over 10 days of storage. The difference in colors could be attributed to the migration of anthocyanins and other phenols from BPP into the simulants, as these active compounds impart color changes at different pH values. Luchese et al. [1] also showed similar coloration upon migration of active compounds from cassava starch films, which they attributed to the formation of the orange/purple flavylium cation under acidic conditions (pH 1.0–3.0). At pH > 4.5, colorless chalcones have been reported to be formed, while under basic conditions (pH > 7), quinoidal base has been reported to produce a blue color [46]. Peaks at 280 nm were observed in both simulants, with much greater peaks in aqueous simulant acetic acid, as shown in Figure 5A,B. This peak is observed due to the migration of aromatic compounds when active component of blueberry pomace were release, which increased with the concentration of acetic acid. Furthermore, another peak at 530 nm was observed only in acetic acid, which could also be attributed to the release of anthocyanins. Various factors such as chemical composition, matrix structure and surrounding media have been reported to influence the diffusion of active compounds from starch-based films [47].

## 5. Conclusions

The thermal, optical and physicochemical properties of edible packaging films produced from different starch sources and with added blueberry pomace powder (BPP) were investigated. Our results showed the potential of developing starch-based films with added blueberry. The films containing higher BPP content had higher UV absorption, which could absorb UV light and protect packaged food content from UV deterioration. Potato and tapioca starch films depicted higher UV absorbance than green bean, corn and sweet potato films. Tapioca starch films were more glossy on the support side, whereas green bean and potato films were more glossy on the air side than other starch films, with glossiness generally decreasing with increasing BPP content. Corn starch films were less glossy, resulting in better printability, and also displayed higher tensile strength than other films. The water vapor transmission rates of the tapioca starch films were highest amongst the various starches used in the study. The glass transition temperatures of all films increased with added BPP content. The feasibility of using these films for the food packaging of aqueous foods was also demonstrated through migration assays, wherein the BPP-enriched starch films were found to allow the higher migration of active phenolics and antioxidants into aqueous food simulants, as compared to fatty food simulants, with corn films showing more migration, followed by tapioca, sweet potato, green bean and potato starch films. Such active packaging with edible films can be used for various food products, especially low to intermediate moisture foods, wherein the active transport of the blueberry phytochemicals can help to improve the quality. 

## Figures and Tables

**Table 1:** The total UV absorption of films made from different types of starch with different starch and blueberry pomace levels.

	Starch Type *				
BPP (%)	Corn	Potato	Green Bean	Sweet Potato	Tapioca
0	58.7 ± 4.4 ^a,1^	114.4 ± 4.4 ^a,2^	50.3 ± 1.9 ^a,1^	60.8 ± 3.4 ^a,1^	132.2 ± 1.3 ^a,2^
0.4	86.3 ± 1.4 ^a,1^	118.7 ± 8.3 ^a,1^	64.3 ± 2.9 ^a,2^	48.3 ± 3.8 ^a,2^	117.4 ± 4.7 ^a,1^
0.8	164.1 ± 1.7 ^b,1^	45.7 ± 18.5 ^b,2^	150.9 ± 1.6 ^b,1^	72.7 ± 3.8 ^b,2^	111.8 ± 2.1 ^a,2^

* Data represented as means ± standard deviations; ^a–b^ Means followed by the same letter in a column are not significantly different; ^1–2^ Means followed by the same number in a row are not significantly different.

**Table 2:** The thickness and transparency value of films made from different types of starch with different starch and BPP levels. Data points represent the means ± standard deviations of three replicates.

	Thickness (mm)				
BPP (%)	Corn	Potato	Green Bean	Sweet Potato	Tapioca
0	0.111 ± 0.01 ^a,1^	0.111 ± 0.09 ^a,1^	0.090 ± 0.02 ^a,1^	0.121 ± 0.04 ^a,1^	0.130 ± 0.06 ^a,1^
0.4	0.118 ± 0.02 ^a,1^	0.118 ± 0.04 ^a,1^	0.128 ± 0.01 ^a,1^	0.109 ± 0.09 ^a,1^	0.120 ± 0.01 ^a,1^
0.8	0.131 ± 0.01 ^a,1^	0.131 ± 0.05 ^a,1^	0.123 ± 0.01 ^a,1^	0.132 ± 0.03 ^a,1^	0.127 ± 0.02 ^a,1^
	**Transparency**				
0	1.02 ± 0.05 ^a,1^	0.53 ± 0.02 ^a,2^	0.75 ± 0.10 ^a,1^	0.63 ± 0.04 ^a,2^	0.47 ± 0.03 ^a,2^
0.4	0.71 ± 0.10 ^b,1^	0.63 ± 0.01 ^a,1^	0.54 ± 0.02 ^a,1^	0.64 ± 0.07 ^a,1^	0.59 ± 0.03 ^a,1^
0.8	0.74 ± 0.07 ^b,1^	0.48 ± 0.17 ^a,1^	0.64 ± 0.04 ^a,1^	0.65 ± 0.02 ^a,1^	0.63 ± 0.03 ^a,1^

^a–b^ Means ± standard deviations followed by the same letter in a column are not significantly different; ^1−2^ Means followed by the same number in a row are not significantly different.

**Table 3:** The gloss of the support (**A**) and air side (**B**) films made from different types of starch with different starch and BPP levels.

(A)					
BPP (%)	Corn	Potato	Green Bean	Sweet Potato	Tapioca
0	42.1 ± 1.6 ^a,1^	42.0 ± 4.8 ^a,1^	49.0 ± 1.3 ^a,1^	42.8 ± 2.5 ^a,1^	74.1 ± 2.8 ^a,2^
0.4	44.7 ± 1.8 ^a,1^	57.5 ± 3.9 ^a,2^	40.5 ± 2.0 ^a,1^	42.0 ± 9.9 ^a,1^	43.1 ± 1.8 ^b,1^
0.8	36.3 ± 3.9 ^b,1^	34.5 ± 2.8 ^a,1^	43.4 ± 3.5 ^a,1^	43.2 ± 2.0 ^a,1^	37.5 ± 1.2 ^c,1^
**(B)**					
**BPP (%)**	**Corn**	**Potato**	**Green Bean**	**Sweet Potato**	**Tapioca**
0	18.6 ± 1.6 ^a,1^	54.0 ± 1.8 ^a,2^	66.6 ± 3.7 ^a,2^	34.3 ± 9.1 ^a,3^	38.5 ± 1.4 ^a,3^
0.4	26.0 ± 6.2 ^a,1^	76.9 ± 9.6 ^b,2^	42.5 ± 1.6 ^a,3^	50.5 ± 1.1 ^a,3^	51.8 ± 5.0 ^a,3^
0.8	25.2 ± 0.5 ^a,1^	79.5 ± 1.2 ^b,2^	53.2 ± 0.7 ^a,3^	55.6 ± 7.5 ^a,3^	43.6 ± 7.9 ^a,3^

^a–b^ Means ± standard deviations followed by the same letter in a column are not significantly different; ^1−3^ Means followed by the same number in a row are not significantly different.

**Table 4:** (A). The water vapor transmission rate (WVTR) (A) of films made from different types of starch with different starch and BPP levels ($\mathrm{unit}:\;g\cdot {\mathrm{cm}}^{-2}\cdot h^{-1})$, (B). The solubility of films made from different types of starch with different starch and BPP levels *.

	(A)				
	WVTR				
BPP (%)	Corn	Potato	Green Bean	Sweet Potato	Tapioca
0	0.00170 ^a,1^	0.00055 ^a,1^	0.00170 ^a,1^	0.00125 ^a,1^	1.36000 ^a,2^
0.4	0.00115 ^a,1^	0.00065 ^a,1^	0.00170 ^a,1^	0.00285 ^a,1^	0.59649 ^a,2^
0.8	0.00105 ^a,1^	0.00070 ^a,1^	0.00300 ^a,1^	0.00115 ^a,1^	2.60870 ^a,2^
	**(B)**				
**BPP**	**Solubility**				
0	29.2% ^a,1^	42.0% ^a,1^	37.0% ^a,1^	31.6% ^a.1^	33.6% ^a,1^
0.4	36.6% ^a,1^	37.1% ^a,1^	31.5% ^a,1^	28.8% ^a,1^	32.6% ^a,1^
0.8	24.1% ^a,1^	32.5% ^a,2^	26.6% ^b,1^	32.1% ^a,2^	35.3% ^a,2^

* Standard deviations not reported as values were less than 1%. ^a–b^ Means ± standard deviations followed by the same letter in a column are not significantly different; ^1−2^ Means followed by the same number in a row are not significantly different.

**Table 5:** The thermal properties of all the films measured using DSC data analysis.

BPP (%)	Corn	Potato	Green Bean	Sweet Potato	Tapioca
The Glass Transition Temperature, Tg (°C)					
0	129.6 ^a^	143.1 ^a^	132.4 ^a^	124.8 ^a^	129.6 ^a^
0.4	145.7 ^a^	134.0 ^a^	141.6 ^a^	143.9 ^a^	147.4 ^a^
0.8	136.7 ^a^	143.3 ^a^	151.5 ^a^	137.7 ^a^	140.6 ^a^
**Enthalpy from the 1st Peak, Δ*H*^1^ (J/g)**					
0	30.6 ^a^	27.4 ^a^	42.2 ^a^	29.7 ^a^	26.3 ^a^
0.4	27.7 ^a^	23.1 ^a^	32.9 ^a^	29.1 ^a^	27.5 ^a^
0.8	27.3 ^a^	26.4 ^a^	17.6 ^a^	37.5 ^a^	31.9 ^a^
**Enthalpy from the 2nd Peak, Δ*H*^2^ (J/g)**					
0	50.9 ^a^	54.7 ^a^	73.1 ^a^	56.6 ^a^	48.6 ^a^
0.4	52.0 ^a^	48.1 ^a^	54.5 ^a^	54.1 ^a^	43.7 ^a^
0.8	42.0 ^b^	43.3 ^a^	38.4 ^b^	61.3 ^a^	49.1 ^a^
**Melting Temperatures from the First Peak, Tm^1^ (°C)**					
0	175.5 ^a^	175.2 ^a^	173.1 ^a^	174.2 ^a^	173.9 ^b^
0.4	175.0 ^a^	176.7 ^a^	172.6 ^a^	175.9 ^a^	177.1 ^b^
0.8	178.2 ^a^	179.9 ^a^	180.3 ^b^	172.6 ^a^	175.2 ^b^
**Melting Temperatures from the Second Peak, Tm^2^ (°C)**					
0	234.7 ^a^	242.9 ^a^	204.8 ^a^	238.6 ^a^	227.3 ^a^
0.4	240.2 ^a^	236.1 ^a^	214.3 ^a^	212.1 ^a^	237.9 ^a^
0.8	235.2 ^a^	236.2 ^a^	239.3 ^a^	226.4 ^a^	224.9 ^a^

^a–b^ Means ± standard deviations followed by the same letter in a column are not significantly different.
